# EMOTIONAL RESPONSES TO SUPPORT FROM VOICE ASSISTANTS WITHIN AN ECOLOGY OF SMART DEVICES

**DOI:** 10.1093/geroni/igae098.2117

**Published:** 2024-12-31

**Authors:** Shannon Mejia, Wan Wen, Smit Desai, Brandon Snipe, Otniel Fernandez, Sungjae Hong, Jessie Chin

**Affiliations:** University of Illinois Urbana-Champaign, Urbana, Illinois, United States; University of Illinois Urbana-Champaign, Champaign, Illinois, United States; University of Illinois Urbana-Champaign, Champaign, Illinois, United States; University of Illinois Urbana-Champaign, Champaign, Illinois, United States; University of Illinois Urbana-Champaign, Champaign, Illinois, United States; University of Illinois Urbana-Champaign, Champaign, Illinois, United States; University of Illinois Urbana-Champaign, Champaign, Illinois, United States

## Abstract

Smart-environments deploy an ecology of smart devices such as voice-assistants (VA), telehealth portals, and smart-fireplaces to support aging in place by providing assistance, remote-assessment, and restoration. Although asking for support is a foundational task within a smart environment, the socioemotional implications of asking smart devices for assistance is largely undefined. This study (N=51, observations=204, age:50-80, 54%female), conducted within a simulated “home office,” employed a 2x2 (fireplace vs. not × neurologist remote-assessment vs. not) within-subjects experiment to test emotional responses to requests for VA assistance under varying smart ecologies. In each condition, a simulated VA instructed participants to play a game and report affect by endorsing adjectives representing their feelings. Participant-initiated requests for VA assistance were extracted from transcripts. Participants’ beliefs in our simulation were evaluated during post-interviews to determine whether interactions were perceived as genuine with a VA (VA-True; 54%) or with a human through a speaker (VA-Human). Multilevel-models indicated higher negative affect (emotional cost) when participants requested VA assistance, and this cost was less pronounced when the smart-fireplace was activated. Cross-level interactions by beliefs differentiated emotional costs of asking for assistance. In general, compared to the VA-Human group, the emotional cost of asking for assistance was less pronounced for the VA-True group. However, while emotional cost was reduced for the VA-Human group when remote-assessment or fireplace was activated, emotional cost was amplified under these conditions for the VA-True group. Findings are situated within life-span perspectives of control and dependency scripts to inform the development of smart-ecologies to support aging-well.

